# Combined Multilayered Amniotic Membrane Graft and Fibrin Glue as a Surgical Management of Limbal Dermoid Cyst

**DOI:** 10.3390/jcm14020607

**Published:** 2025-01-18

**Authors:** Maria Poddi, Vito Romano, Alfredo Borgia, Floriana Porcaro, Carlo Cagini, Marco Messina

**Affiliations:** 1 Ophthalmology Section, Department of Medicine and Surgery, University of Perugia, 06132 Perugia, Italy; maria-poddi@libero.it (M.P.); carlo.cagini@unipg.it (C.C.); 2 Eye Unit, Department of Medical and Surgical Specialties, Radiological Sciences, and Public Health, University of Brescia, Viale Europa 15, 25123 Brescia, Italy; vito.romano@unibs.it; 3 Cornea Unit, Mons. Dimiccoli Hospital, 70051 Barletta, Italy; alfredo.borgia@aslbat.it; 4 Consorzio Sannio Tech, 82030 Apollosa, Italy; floriana.porcaro@gmail.com

**Keywords:** corneal dermoid, amniotic membrane, mitomycin C, fibrin glue

## Abstract

**Background/Objectives:** To report the cosmetic, clinical, and visual outcomes of a combined surgical approach for treating a corneal/limbal dermoid using excision and a three-layered amniotic membrane graft with fibrin glue. **Methods:** An 18-year-old female presented with impaired vision and ocular discomfort caused by a prominent dome-shaped limbal congenital dermoid on the inferotemporal cornea, resulting in a significant aesthetic concern. A full assessment, including refraction, best-corrected visual acuity (BCVA), corneal topography, aberrometry and anterior segment OCT (AS-OCT) was conducted to plan the surgical approach. The dermoid was excised under peribulbar anaesthesia using manual lamellar dissection, followed by the application of 0.02% Mitomycin C and a multilayered amniotic membrane graft with fibrin glue. A bandage contact lens was applied and removed after three weeks, with postoperative treatment including topical antibiotics and steroids. Follow-ups were conducted on day 1, at 1 week, 3 weeks, 2 months, 6 months, 1 year, and 2 years. **Results:** Histopathological examination confirmed the mesoblastic nature of the lesion. Significant improvements in BCVA and ocular symptoms were observed. Corneal topography showed ocular surface regularization with reduction of high order aberrations and point spread function. AS-OCT showed complete integration of the amniotic membrane, with full epithelial coverage of the defect. The healing process was uneventful and the ocular surface remained stable throughout the entire follow-up, without complications or recurrence. **Conclusions:** This approach of dermoid excision, multilayered amniotic membrane and fibrin glue restored vision effectively, with notable improvements in ocular surface and cosmetic outcomes, without recurrence over two years.

## 1. Introduction

Ocular dermoids are benign choristomas of ectodermal and mesodermal origin that can occur on the cornea, limbus, and/or conjunctiva [1]. Their incidence among newborns is approximately 1 to 3:10,000 and they are the most common congenital orbital lesion accounting for 25% of invasive orbital lesions [2]. These lesions typically present as yellowish to whitish dome-shaped masses, occasionally displaying sebaceous material on their surface, and are characterized by the absence of a well-developed vascular network [3]. An anatomical classification based on the site and the extent of the affected cornea discerns 3 grades. Grade I is a superficial dermoid < 5 mm in diameter localized at corneal limbus, Grade II refers to a large lesion covering almost the whole cornea going deep up, but sparing the Descemet’s membrane. The least common is Grade III, an extensive dermoid involving the entire cornea both on the surface and in depth, potentially affecting the iris epithelium as well [3]. Although ocular dermoids generally exhibit a slow growth pattern, they may lead to visual impairment due to induced irregular astigmatism and anisometropic amblyopia, particularly in cases involving extensive lesions [4,5]. Advanced cases may result in ocular discomfort and irritation, with the potential for Dellen formation [4]. Cosmetic concerns associated with ocular dermoids, including even small lesions, are also significant [6]. The standard of care for ocular dermoids is surgical intervention. In detail, surgical indications concern Grade II and III dermoids or even Grade I when high astigmatism or a considerable aesthetic impact happen to occur [7,8,9]. A spectrum of surgical procedures has been documented, ranging from simple surgical excision to lamellar keratoplasty with a SMILE-extracted lenticule and/or penetrating keratoplasty with corneal-limbal-scleral donor graft transplantation [6,7,8,9,10,11,12,13]. This case report outlines the visual and cosmetic outcomes of a Grade I limbal dermoid treated with a combined surgical approach that included excision, application of a three-layered amniotic membrane (AM) and Mitomycin C (0.02%) to reduce the risk of postoperative recurrence [5]. The simple excision alone carries intrinsic risks of corneal opacification, persistent epithelial defects, and pseudo-pterygium formation [9,10,11,12,13]. Conversely, the use of an amniotic membrane graft promotes re-epithelialization while minimizing inflammation, vascularization and scarring [10,12].

## 2. Materials and Methods

**Figure 1:**
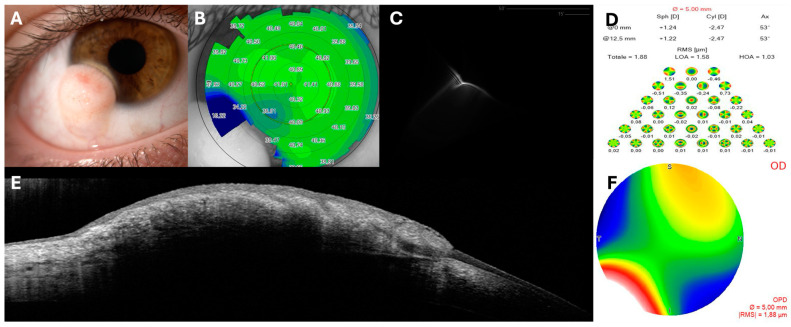
Preoperative slit lamp photograph of inferotemporal limbal dermoid (**A**). Sirius topography image of anterior tangential curvature revealing a significant irregularity of the ocular surface (**B**). Aberrometric assessments showing Point Spread Function (PSF) with Strehl Ratio (**C**) and whole corneal aberrations (**D**,**F**). Heidelberg Spectralis anterior segment optical coherence tomography (AS − OCT cornea module) scan showing the hyperreflective lesion without a clear evidence of its depth (**E**).

An 18-year-old female was referred to our cornea clinic due to visual impairment and ocular discomfort caused by a Grade I dome-shaped congenital dermoid in the inferotemporal cornea of her right eye (Figure 1). The patient reported significant distress regarding the impact of the lesion on her personal and social life, primarily related to aesthetic concerns. During her initial visit, a comprehensive refraction assessment and evaluation of best-corrected visual acuity (BCVA) were conducted, revealing severe impairment due to consistently high irregular astigmatism (Table 1). Notably, no amblyopia was detected. Corneal topography with aberrometry was performed using the Scheimpflug-Placido Sirius topographer (CSO, Florence, Italy), in conjunction with a thorough slit-lamp examination (Digital Slit Lamp SL-9900, CSO, Florence, Italy) and an epithelial map (MS-39, CSO, Florence, Italy) (Figure 1). Spectral domain anterior segment optical coherence tomography (AS-OCT, HRA II, Heidelberg Engineering GmbH, Dossenheim, Germany; MS-39, CSO, Florence, Italy) was employed for high-resolution imaging to detect the depth of the lesion, which was unclear, and for follow-up evaluations. Following an in-depth discussion of therapeutic options, the surgical approach involving excision, administration of Mitomycin C 0.02% and application of a multilayered amniotic membrane fixed by three 10-0’ nylon sutures and fibrin glue, was deemed the most appropriate course of action. Follow-ups were conducted on day 1, at 1 week, 3 weeks, 2 months, 6 months, 1 year, and 2 years.

### Surgical Procedure

The patient was treated under peribulbar anaesthesia with mepivacaine hydrochloride 2% and levobupivacaine 0.5%. A Westcott scissors-assisted peritomy of the conjunctiva was performed around and on the dermoid, which measured approximately 5 mm × 8 mm, to free it from the surrounding tissue, after the injection of 0.1 cc of preservative-free lidocaine 0.75% with epinephrine. A manual dissection of the lesion with a crescent blade was performed, starting from the base of the dermoid and progressing from the sclera towards the cornea, showing a significant scleral and corneal defect. A beaver blade knife was subsequently used to gently excise any residual dermoid fibers. Once the corneal-scleral surface appeared smooth, 0.02% Mitomycin C was applied for 2 min followed by copious irrigation with balanced salt solution (BSS) to prepare the surgical bed for the application of a multilayered amniotic membrane. Three layers of the same size of amniotic membrane were assembled in a sandwich pattern with fibrin glue (Tisseel^®^, Baxter Inc., Deerfield, IL, USA) between them, ensuring the stromal side faced the corneal surface and the epithelial side faced upward, all layers behaving as a graft. The last layer of the membrane was then trimmed to match the dimensions of the measured corneal defect, achieving complete filling of the corneal depth while leaving a slight step in order to promote the growth of the host epithelium. Three 10-0’ nylon stitches were then applied. The recessed conjunctival tissue was repositioned adjacent to the edge of the amniotic membrane using fibrin glue. A bandage contact lens (BCL) was inserted postoperatively and removed, along with the stitches, at the three-week follow-up visit. Post-surgical treatment included moxifloxacin 5 mg/mL eye drops four times daily for three weeks (Moxidrop, FB-Vision S.p.A, Via San Giovanni Scafa, 63074 San Benedetto del Tronto, AP, Italy) and preservative-free dexamethasone sodium phosphate 1 mg/mL eye drops twice daily for a month (Dexavison, FB-Vision S.p.A, Via San Giovanni Scafa, 63074 San Benedetto del Tronto, AP, Italy). The patient was re-evaluated postoperatively on day 1, at 1 week, 3 weeks, 2 months, 6 months, 1 year, and 2 years with full assessments.

## 3. Results

**Figure 2:**
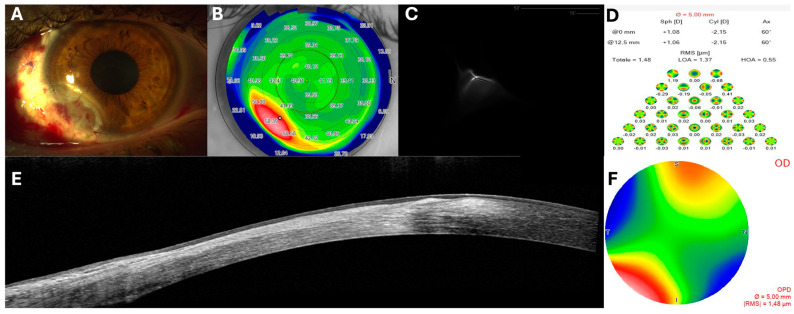
1 week postoperative slit lamp image showing the multilayered amniotic membranes graft perfectly covering the defect after the dermoid removal (**A**). Sirius topography images revealing a significant improvement in the regularity of the ocular surface (**B**). Aberrometric assessment with a substantial reduction in total corneal aberrations including both LOAs and HOAs, along with a notable improvement in PSF and Strehl ratio (**C**,**D**,**F**). Heidelberg Spectralis OCT images demonstrating a complete integration (graft) of the three-layered amniotic membrane into the corneoscleral surface (**E**).

**Figure 3:**
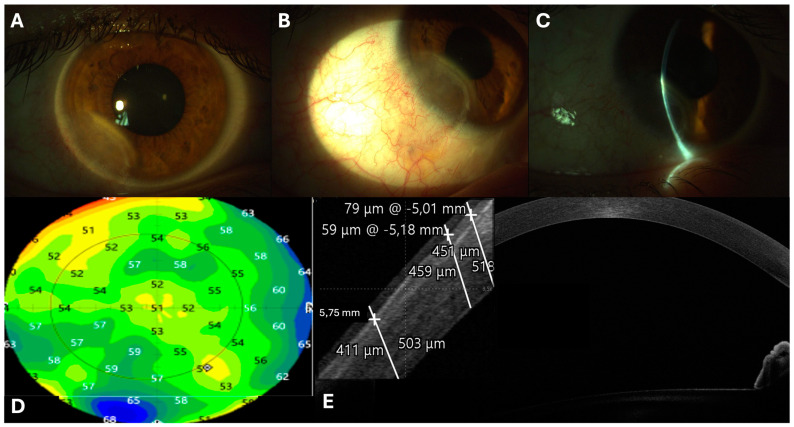
Slit lamp images taken two years post-surgery showing no signs of recurrence (**A**,**B**), proper corneal thickness (**C**), and no evidence of neovascularization or significant scarring (**A**,**B**). The epithelial map (**D**) and OCT scans (**E**) show a compensatory epithelial thickening in the inferotemporal cornea (between 63 µm and 68 µm).

The patient’s recovery following surgery was remarkably uneventful, with no reported discomfort on the ocular surface the day after the procedure. The bandage contact lens (BCL) remained securely in place and there was no significant inflammation. The three layers of amniotic membrane were stable and properly positioned, promoting uniform epithelial growth and sutures appeared meticulously aligned (Figure 2A). Follow-up evaluations at one week and three weeks demonstrated continued enhancement in the regularity of the ocular surface (Figure 2B), with the amniotic membrane fully integrated into the corneoscleral surface as seen at the AS-OCT scans (Figure 2E). Epithelial compensation was clearly evident in the epithelial mapping and MS-39 OCT scans (Figure 3D,E). Topographic assessments revealed significant enhancement in surface regularity along with reduction in both lower-order aberrations (LOAs) and higher-order aberrations (HOAs) (Figure 1 and Figure 2, Table 1). Additionally, there was a substantial improvement in point spread function (PSF) over time, as indicated by an increased Strehl Ratio (Table 1, Figure 2B–D,F). Best-corrected visual acuity (BCVA) showed a progressive upgrading, achieving stabilization by three months postoperatively (Table 1). Throughout the follow-up period no complications were encountered, and by the two-year follow-up, the patient showed no signs of recurrence, significant corneal scarring and/or neovascularization (Figure 3A–C).

## 4. Discussion

Epi-bulbar dermoid can be treated through various conservative and/or surgical approaches. Grade I dermoids can be managed medically prescribing spectacles and treating the potential related amblyopia [13]. So far, although there is no univocal and certain surgical indication for Grade I dermoids, irregular astigmatism and cosmetic concerns are the two major indications for surgery [14]. Grade II and III dermoids are always treated surgically as they can lead to refractive or occlusive amblyopia. Different surgical methods for dermoids removal have been described depending on the depth, size and location of the lesion ranging from simple excision to lamellar keratoplasty with a SMILE-extracted lenticule and/or penetrating keratoplasty with corneal-limbal scleral donor graft transplantation [6,15]. Simple dermoid excision carries a significant risk of persistent corneal defect, pseudo-pterygium occurrence, corneal scarring, neovascularization and micro-perforations, also resulting in undesirable cosmesis [12,16]. Other procedures encounter different complications as opacification of the graft, haze in the graft, graft rejection, limbal stem cell deficiency, interface neovascularization and steroid-induced glaucoma [17]. The use of amniotic membrane for corneal and conjunctival reconstruction has been widely described in literature as it successfully heals epithelial defects and decreases ulcer size in infectious corneal ulcer, therefore helping in the filling of stromal and epithelial defect after limbal dermoid surgery [18]. Another mechanism of action of the amniotic membrane regards its ability to reduce inflammation through a significant antiprotease activity, mitigating scarring, vascularization, and recurrence of lesions [18]. The orientation of the amniotic membrane is crucial; when the AM is intended to be used as a graft (incorporated into the host tissue) and acts as a substrate or scaffold for epithelial cells to grow, then the basement has to be placed membrane side up. When it is applied as a patch, it is used as a cover or a biological bandage “contact lens” protecting the underlying healing epithelial surface [19,20,21]. Given that the combined approach based on the deep lamellar excision followed by multilayered amniotic membrane transplantation was described in literature, we got a hint from this point and adjusted this method in order to gain a suitable surgery and a proper outcome for our patient [5]. Limbal stem cell deficiency and pseudo-pterygium are common postoperative complications related to corneal dermoid removal, thus we opted for intraoperative use of Mitomycin C 0.02% as an adjunct [22,23,24,25]. Mitomycin C is an alkylating agent that inhibits RNA and DNA synthesis leading to cytotoxic effects and fibroblasts apoptotic cell death [22,23,24]. Furthermore, to ensure a proper adhesion and stability of the amniotic membrane, we adopted three 10-0’ nylon sutures and fibrin glue. This combined approach yielded excellent results in terms of topographic regularity and epithelial thickness, effectively addressing both cosmetic and visual outcomes. While this study acknowledges the inherent limitations of a single case report, it is noteworthy that current literature lacks comprehensive evaluations of two-year outcomes following limbal dermoid surgery using our combined approach and analysed throughout the entire follow-up with multiple diagnostic tools such as AS-OCT, corneal topography, aberrometry and epithelial map.

## 5. Conclusions

Our findings underscore the safety and efficacy of a combined approach in achieving optimal topographic regularity and epithelial thickness, while simultaneously enhancing cosmetic and visual outcomes for patients undergoing limbal dermoid surgery over a two-year follow-up. This is the first study to provide a detailed instrumental analysis of the functional and anatomical outcomes resulting from this combined surgical approach. The integration of advanced imaging techniques, including AS-OCT, topography, aberrometry, and epithelial mapping, provides a thorough assessment of postoperative results that has not been previously documented in the literature. Future research involving larger cohorts and longitudinal follow-up is warranted to validate these findings and further elucidate the long-term implications of this surgery.

## Figures and Tables

**Table 1:** Synoptic overview of changes in refractive and aberrometric parameters.

	BCVA (LogMar)	Cylindrical Power (D)	Axis of Cylinder	Spherical Equivalent (D)	Root Means Square (µm)	Strehl Ratio	LOA (µm)	HOA (µm)
Preoperative	0.1	2.10	55°	−1.50	1.88	0.1192	1.58	1.03
Postoperative	0	1.49	60°	−1.00	1.48	0.1353	1.37	0.55

BCVA: Best-corrected visual acuity; D: Diopters; LOA: Lower order aberrations; HOA: Higher order aberrations.

## Data Availability

The data presented in this study are stored in the hospital registry.

## References

[1] Jeong J., Song Y.J., Jung S.I., Kwon J.W. (2015). New surgical approach for limbal dermoids in children: Simple excision, corneal tattooing, and sutureless limboconjunctival autograft. Cornea.

[2] Arce Gonzalez M.d.R., Navas A., Haber A., Ramírez-Luquín T., Graue-Hernández E.O. (2013). Ocular dermoids: 116 consecutive cases. Eye Contact Lens.

[3] AlGhadeer H., Kirat O., Vargas J., AlBadr L., Khandekar R. (2023). Visual and surgical outcomes of limbal dermoid excision at a tertiary care eye hospital. Eur. J. Ophthalmol..

[4] Zhong J., Wang W., Li J., Wang Y., Hu X., Feng L., Ye Q., Luo Y., Zhu Z., Li J. (2022). Effects of perceptual learning on deprivation amblyopia in children with limbal dermoid: A randomized controlled trial. J. Clin. Med..

[5] Lang S.J., Böhringer D., Reinhard T. (2014). Surgical management of corneal limbal dermoids: Retrospective study of different techniques and use of Mitomycin C. Eye.

[6] Liu J.-L., Ji J.-Y., Ye Q., Wei L.-Q., Zhong X., Jiang L.-Z., Zeng J. (2023). Treatment of corneal dermoid with lenticules from small incision lenticule extraction surgery: A surgery assisted by fibrin glue. Int. J. Ophthalmol..

[7] Scott J.A., Tan D.T.H. (2001). Therapeutic lamellar keratoplasty for limbal dermoids. Ophthalmology.

[8] Shen Y.D., Chen W.L., Wang I.J., Hou Y.C., Hu F.R. (2005). Full-thickness central corneal grafts in lamellar keratoscleroplasty to treat limbal dermoids. Ophthalmology.

[9] Wan Q., Tang J., Han Y., Ye H. (2019). Surgical Treatment of Corneal Dermoid by Using Intrastromal Lenticule Obtained from Small-Incision Lenticule Extraction. Int. Ophthalmol..

[10] Cho W.-H., Sung M.-T., Lin P.-W., Yu H.-J. (2018). Progressive Large Pediatric Corneal Limbal Dermoid Management with Tissue Glue-Assisted Monolayer Amniotic Membrane Transplantation. Medicine.

[11] Hao Y., Zhu B., Wu T., Guo X. (2024). Sutureless lamellar keratoplasty with lenticule from small incision lenticule extraction for treating limbal dermoid: A case report. Exp. Ther. Med..

[12] Pirouzian A., Ly H., Holz H., Sudesh R.S., Chuck R.S. (2011). Fibrin-glue assisted multilayered amniotic membrane transplantation in surgical management of pediatric corneal limbal dermoid: A novel approach. Graefes Arch. Clin. Exp. Ophthalmol..

[13] Cui Y., Yin S., Yin X., Liu Y., Zhao B. (2021). Removal of a congenital corneal dermoid through tumor excision and lamellar keratoplasty in a young child: A case report. Medicine.

[14] Robb R.M. (1996). Astigmatic refractive errors associated with limbal dermoids. J. Pediatr. Ophthalmol. Strabismus.

[15] Panda A., Ghose S., Khokhar S., Das H. (2002). Surgical outcomes of epibulbar dermoids. J. Pediatr. Ophthalmol. Strabismus.

[16] Panton R.W., Sugar J. (1991). Excision of limbal dermoids. Ophthalmic Surg..

[17] Mader T.H., Stulting D. (1998). Technique for the removal of limbal dermoids. Cornea.

[18] Gomes J.A., Romano A., Santos M.S., Dua H.S. (2005). Amniotic membrane use in ophthalmology. Curr. Opin. Ophthalmol..

[19] Tseng S.C., Espana E.M., Kawakita T., Di Pascuale M.A., Li W., He H., Liu T.S., Cho T.H., Gao Y.Y., Yeh L.K. (2004). How does amniotic membrane work?. Ocul. Surface J..

[20] Yildiz E.H., Nurozler A.B., Ozkan Aksoy N., Altiparmak U.E., Onat M., Karaguzel H. (2008). Amniotic membrane transplantation: Indications and results. Eur. J. Ophthalmol..

[21] Messina M., Poddi M., De Santi N., Bianchi E., Cagini C. (2024). Combined superficial keratectomy, alcohol delamination and amniotic membrane patch with fibrin glue in Salzmann nodular degeneration. Eur. J. Ophthalmol..

[22] Raiskup F., Solomon A., Landau D., Ilsar M., Frucht-Pery J. (2004). Mitomycin C for pterygium: Long term evaluation. Br. J. Ophthalmol..

[23] Grossniklaus H.E., Aaberg T.M. (1997). Mitomycin C Treatment of Conjunctival Intraepithelial Neoplasia. Am. J. Ophthalmol..

[24] Cheng H.-C., Tseng S.-H., Kao P.-L., Chen F.K. (2001). Low-dose Intraoperative Mitomycin C as Chemoadjuvant for Pterygium Surgery. Cornea.

[25] Kate A., Basu S. (2022). A Review of the Diagnosis and Treatment of Limbal Stem Cell Deficiency. Front. Med..

